# Follicle-Stimulating Hormone Receptor and Estrogen Receptor Gene Polymorphisms in Women With Discordant Follicle-Stimulating Hormone and Anti-Mullerian Hormone Levels

**DOI:** 10.7759/cureus.60446

**Published:** 2024-05-16

**Authors:** Görkem Aktaş, Mete Bertizlioglu, Setenay Arzu Yılmaz, Ayse Gül Kebapcılar, Çetin Çelik, Özlem Seçilmiş

**Affiliations:** 1 Obstetrics and Gynecology, Dr. Ali Kemal Belviranlı Gynaecology and Children's Hospital, Konya, TUR; 2 Obstetrics and Gynecology, Konya City Hospital, Konya, TUR; 3 Obstetrics and Gynecology, Selcuk University Medicine, Konya, TUR; 4 Obstetrics and Gynecology, Medova Hospital, Konya, TUR

**Keywords:** ovarian reserve tests, anti-mullerien hormone, estrogen hormone receptor, follicle-stimulating hormone receptor, ovarian reserve

## Abstract

Objective

This study aimed to investigate follicle-stimulating hormone receptor (FSHR) polymorphisms (Thr307Ala and Asn680Ser), estrogen receptor 1 (ESR1) polymorphisms (PvuII and XbaI), and ESR2 polymorphisms (RsaI and AluI) in Turkish women with follicle-stimulating hormone (FSH) and anti-Mullerian hormone (AMH) discordance.

Method

Genotyping was performed in 60 patients aged 21-35 with FSH-AMH discordance and/or low ovarian reserve and 20 age-matched controls with normal FSH and AMH levels. The patients were investigated in four groups of 20 women according to their FSH and AMH levels. Groups 1, 2, 3, and 4 were as follows: normal FSH and low AMH levels, normal AMH and high FSH levels, high FSH and low AMH levels, and normal FSH and AMH levels. Genomic DNA was obtained from 3 cc peripheral blood, and polymorphisms were analyzed using TaqMan genotyping assays. Relations between groups of categorical variables were analyzed with a chi-square test. Differences between the groups were assessed using a student’s t-test or Mann-Whitney U test.

Results

Women with discordant FSH and AMH levels (group 1 and group 2) were not statistically different from women with concordant FSH and AMH levels (group 3 and group 4) in terms of FSHR, ESR1, and ER2 single nucleotide polymorphisms (SNPs). Body mass index (BMI) was statistically significant between groups 1 and 2 as well as groups 2 and 3 (p = 0.004).

Conclusions

This study showed that FSHR, ESR1, and ESR2 SNPs have not had any effect on AMH-FSH discordance in reproductive age Turkish women.

## Introduction

Ovarian reserve refers to the size and quality of the ovarian follicular pool and the potential of the ovaries to respond to exogenous gonadotropin stimulation [1]. The quality and quantity of ovarian reserve are influenced by genetic and demographic factors such as age and body mass index (BMI) [1,2]. Ovarian reserve can be predicted biochemically and ultrasonographically by ovarian tests. Serum follicle-stimulating hormone (FSH) and anti-Mullerian hormone (AMH) are the most commonly used biochemical tests [3].

Although FSH and AMH are different hormones, there is a general use of concomitant measurement in the assessment of ovarian reserve. With increasing age and diminishing ovarian reserve, AMH levels generally decrease and FSH levels increase concordantly [4]. However, 20%-43% discordance is observed between FSH and AMH [5,6]. Therefore, the interpretation of ovarian reserve tests should be individualized [7,8]. Different phenotypes with altering ovarian reserve test results can be caused by genetic differences called polymorphisms. Many studies have investigated the relationship between single nucleotide polymorphisms (SNPs) and gynecological diseases and aimed to elucidate the differences caused by FSH receptor isoforms. These studies have shown that SNPs may alter exogenous FSH levels, leading to differential responses to controlled ovarian hyperstimulation (COH) [9-11].

The follicle-stimulating hormone receptor (FSHR) gene contains two important SNPs in exon 10 that change two amino acids at the Threonin307Alanine (Thr307Ala) and Asparagine680Serine (Asn680Ser) positions [12]. Alviggi et al. showed that patients with the Ser/Ser genotype were less sensitive to exogenous gonadotropins [13]. König et al. found that Ser/Ser genotype was significantly associated with poor ovarian response to COH [14]. Estrogen receptor (ESR) genetic screening revealed the presence of several polymorphic regions [15]. The most frequently studied are Pvu II and Xba I SNPs [15,16]. Li et al. showed that there was no significant association between polymorphisms in the ESR gene and poor ovarian response [17].

Based on the assumption that feedback mechanisms and sensitive hormonal communication systems in the hypothalamic-pituitary-ovarian axis may be affected by polymorphic individual differences in the genes encoding FSH and estrogen hormones, this study aimed to investigate FSHR (Thr307Ala, Asn680Ser), ESR1 (PvuII and XbaI), and ESR2 polymorphisms (RsaI and AluI) in Turkish women of reproductive age with FSH and AMH discordance.

## Materials and methods

Study subjects and patient selection

This prospective study included women aged 21-35 years who presented to the infertility clinic at Selçuk University Hospital in Konya between January 1, 2019, and January 1, 2020. The study was approved by the Clinical Research Ethical Committee of Selçuk University Faculty of Medicine (approval number: 2020/183). The patients who provided data for the study were invited to participate and signed an informed consent form.

Patients under the age of 21 and above the age of 35, smokers, users of drugs that may affect ovarian function and lipid levels, those with a history of chronic disease, and those receiving hormonal drug therapy in the three months preceding the study were not included in the sample.

Study design

The women were investigated in four groups of 20 patients according to their FSH and AMH levels. The basal levels of FSH/AMH were measured in all women between the second and fourth day of the menstrual cycle.

The cut-off value for AMH was set at 1 ng/mL, and values above this were considered normal; patients with values below this were classified as having low AMH levels. FSH levels were accepted as a limit value of 10 IU/L in patients. Patients with values below this level were grouped as normal FSH levels, and patients with values above this level were included in the high FSH group. Patients with normal FSH and low AMH levels were included in group 1 (n = 20), while group 2 consisted of patients with normal AMH and high FSH values (n = 20) (Group 1 and 2/discordant groups). Group 3 consisted of patients with high FSH and low AMH values (n = 20) (Group 3/low ovarian reserve) The fourth group consisted of the control group with normal FSH and normal AMH values (n = 20) (Group 3/normal). Age and BMI values of the patients were also recorded. Genotyping was performed in 40 women between the ages of 21 and 35 with FSH-AMH discordance, 20 women with low ovarian reserve, and 20 age-matched controls with normal FSH and AMH levels.

Biochemical and genetic analysis

The serum FSH was measured on the Cobas 8000 CORE platform (Roche Diagnostics, Indianapolis, Indiana), which has a solid-phase, competitive chemiluminescent immunometric enzyme immunoassay system. The AMH levels were all measured with the Reprosource AMH assay, a laboratory-developed test, and were performed at a single reference laboratory.

About 3 cc venous blood samples obtained from the patients were stored in an ethylenediaminetetraacetic acid (EDTA) tube at -20°C. SNPs were genotyped using TaqMan assays (Applied Biosystems, Foster City, CA) (Table 1). The 12 uL reaction mix contained 25 ng genomic DNA, 0.25 x stock genotyping assay, and 1 x TaqMan genotyping PCR master mix. Amplification and hybridization were performed using the Applied Biosystems StepOnePlus Real-Time PCR System according to the manufacturer's recommendation.

**Table 1:** Analyzed SNP annotations *TaqMan® SNP Genotyping assay, Applied Biosystems, Foster City, CA. SNP: Single nucleotide polymorphism; ID: Identification card; HGVS: Human genome variation society; FSHR: Follicle-stimulating hormone receptor; ESR: Estrogen receptor.

Gene	rs ID	HGVS	Clinical definition	Assay ID*
FSHR	rs6165	c.919A>G	Ala307Thr	C___2676873_30
	rs6166	c.2039A>G	Asn680Ser	C___2676874_10
ESR1	rs9340799	c.351A>G	XbaI	C___3163591_10
	rs2234693	c.397T>C	PvuII	C___3163590_10
ESR2	rs1256049	c.1082G>A	RsaI	C___7573265_10
	rs4986938	c.1730A>G	AluI	C__11462726_10

Statistical analysis

Statistical Package for the Social Sciences (SPPS) version 25 (IBM Corp., Armonk, NY) was used. Descriptive statistics (mean, standard deviation, median value, minimum, maximum, number, and percentile) were given for categorical and continuous variables. In addition, homogeneity of variances, one of the prerequisites of parametric tests, was checked by the "Levene" test. The normality assumption was checked with "Shapiro-Wilk" test. When the differences between the two groups were to be evaluated, "student's t-test" was used when the prerequisites for parametric tests were met, and the "Mann-Whitney U test" was used when they were not met. One-way analysis of variance (ANOVA) and Tukey's honest significant difference (HSD) test, one of the multiple comparison tests, were used for three or more group comparisons. The Kruskal-Wallis and Bonferroni-Dunn tests, one of the multiple comparison tests, were used when they were not met. The relationship between two continuous variables was evaluated with the Pearson correlation coefficient and Spearman correlation coefficient in cases where parametric test prerequisites were not met. Relationships between groups of categorical variables were analyzed with the chi-square test. In cases where the expected frequencies were less than 20%, the "Monte-Carlo simulation method" was used to include these frequencies in the analysis. Cramer's V and odds ratio is effect size values used for chi-square statistics. The risk ratio value is used for categorical variables consisting of two groups. Since there are four groups in a categorical variable in this study, Cramer's V effect size was used. p < 0.05 level was considered statistically significant.

## Results

A comparison of the clinical data of the study groups showed a statistical difference in BMI measurements between the groups. BMI was highest in group 1 at 27.95 ± 3.137 and lowest in group 2 at 25.05 ± 3.41. A significant difference was observed between groups 1 and 2 as well as between groups 2 and 4 (p = 0.004). Demographic data of the patients are shown in Table 2.

**Table 2:** Demographic data of the groups Data expressed as mean ± SD. ^A, B^: Different letters or letter combinations in the same row indicate a statistically significant difference (p < 0.05). BMI: Body mass index; FSH: Follicle-stimulating hormone; AMH: Anti-Mullerian hormone.

	Group 1: FSH normal, AMH low	Group 2: FSH high, AMH normal	Group 3: FSH normal, AMH normal	Group 4: FSH high, AMH low	p-value
Age	31.3 ± 5.059	30.9 ± 3.37	29.15 ± 3.514	29.85 ± 4.694	0.3492
BMI (kg/m^2^)	27.95 ± 3.137^A^	25.05 ± 3.41^B^	25.45 ± 3.62^A,B^	27.9 ± 2.49^A^	0.0041

There are statistically significant differences in the comparison of FSH values between the groups (p = 0.001). As it was planned to include patients with normal FSH levels in groups 1 and 4 and patients with high FSH levels in groups 2 and 3, this result is statistical evidence that the groups were formed as expected. AMH values also showed a statistically significant difference between the patient groups (p = 0.017). This result shows that groups 1 and 3 had low AMH levels, while groups 2 and 4 had normal AMH levels, thus proving that the groups were formed as expected. The laboratory data of the patients are shown in Table 3.

**Table 3:** Laboratory results of the groups Data expressed as mean ± SD. ^A, B^: Different letters or letter combinations in the same row indicate a statistically significant difference (p < 0.05). FSH: Follicle-stimulating hormone; AMH: Anti-Mullerian hormone.

	Group 1: FSH normal, AMH low	Group 2: FSH high, AMH normal	Group 3: FSH normal, AMH normal	Group 4: FSH high, AMH low	p-value
FSH (mUI/mL)	6.05 ± 1.762^A^	13.27 ± 5.299^B^	6.5 ± 1.39^A^	14.52 ± 2.296^B^	0.001
AMH (ng/mL)	0.67 ± 0.171^A^	2.11 ± 0.959^B^	3.76 ± 4.529^B^	0.54 ± 0.145^A^	0.017

Genotypes and allotypes of FSHR The307Ala (rs6165) SNPs were evaluated in four groups, and no statistically significant difference was observed between the GG genotypes in the comparative analysis of the groups in this study (p = 0.59). GG genotype was highest in group 3 at 35% and lowest in group 2 at 15%. GA genotypes did not show statistically significant differences in the groups (p = 0.763). GA genotype was highest in groups 1 and 2 with 29% and lowest in group 3 with 18%. AA genotypes did not show statistically significant differences in the groups (p = 0.909). In the allotype analysis of rs6165 polymorphism in the FSHR gene in four groups, no statistically significant difference was observed in G and A allotypes. Genotype and allotype data of FSHR gene The307Ala (rs6165) polymorphisms are given in Table 4.

**Table 4:** Genotype and allotype data of FSHR gene The307Ala (rs6165) polymorphism Data expressed as mean ± SD. p < 0.05 is considered significant. FSH: Follicle-stimulating hormone; AMH: Anti-mullerian hormone; FSHR: Follicle-stimulating hormone receptor.

The307Ala (rs6165)	Group 1: FSH normal, AMH low (n%)	Group 2: FSH high, AMH normal (n%)	Group 3: FSH high, AMH low (n%)	Group 4: FSH normal, AMH normal (n%)	p-value
GG	5 (25)	3 (15)	7 (35)	5 (25)	0.659
GA	11 (29)	11 (29)	7 (18)	9 (24)	0.763
AA	4 (18)	6 (27)	6 (27)	6 (27)	0.909
Allotype					
A	21 (27)	17 (22)	21 (27)	19 (24)	0.905
G	19 (23)	23 (28)	19 (23)	21 (26)	0.911

There was no statistically significant difference between groups for the GG genotype of the FSHR Asn680Ser (rs6166) polymorphism (p = 0.801). GA genotypes do not show statistically significant differences in groups (p = 0.837). AA genotype was observed at the highest rate of 35% in group 4, which was selected as the control group, and at the lowest rate of 20% in groups 1 and 3. There was no statistical difference in the comparative analysis of AA genotype between the groups (p = 0.753). In the allotype analysis of rs6166 polymorphism in the FSHR gene in four groups, no statistically significant difference was observed in G and A allotypes. Genotype and allotype data of FSHR gene Asn680Ser (rs6166) polymorphisms are given in Table 5.

**Table 5:** Genotype and allotype data of FSHR gene Asn680Ser (Rs6166) polymorphism Data expressed as mean ± SD. p < 0.05 is considered significant. FSH: Follicle-stimulating hormone; AMH: Anti-mullerian hormone; FSHR: Follicle-stimulating hormone receptor.

Asn680Ser (rs6166)	Group 1: FSH normal, AMH low (n%)	Group 2: FSH high, AMH normal (n%)	Group 3: FSH high, AMH low (n%)	Group 4: FSH normal, AMH normal (n%)	p-value
GG	5 (26)	3 (16)	6 (32)	5 (26)	0.801
GA	11 (27)	12 (29)	10 (24)	8 (20)	0.837
AA	4 (20)	5 (25)	4 (20)	7 (35)	0.753
Allotype					
A	21 (27)	18 (23)	22 (28)	18 (23)	0.886
G	19 (23)	22 (27)	18 (22)	22 (27)	0.890

Haplotype analysis of FSHR gene Rs6165 and Rs6166 SNPs were analyzed in four groups in this study. No statistically significant difference was observed in the comparative analysis of the groups in the haplotypes analyzed. Haplotype data for the FSHR gene rs6165 and rs6166 polymorphisms are shown in Table 6.

**Table 6:** Haplotype data of FSHR gene The307Ala (Rs6165) and Asn680Ser (Rs6166) polymorphisms Data expressed as mean ± SD. p < 0.05 is considered significant. FSH: Follicle-stimulating hormone; AMH: Anti-Mullerian hormone; Follicle-stimulating hormone receptor.

Haplotype	Group 1: FSH normal, AMH low (n%)	Group 2: FSH high, AMH normal (n%)	Group 3: FSH high, AMH low (n%)	Group 4: FSH normal, AMH normal (n%)	p-value
GGAA	1 (14)	0 (0)	5 (71)	1 (14)	0.102
GGGA	4 (44)	2 (22)	2 (22)	1 (11)	0.550
GGGG	0 (0)	1 (25)	0 (0)	3 (75)	0.317
GAGG	2 (29)	3 (43)	0 (0)	2 (29)	0.867
GAGA	5 (23)	6 (27)	7 (32)	4 (18)	0.823
GAAA	4 (44)	2 (22)	0 (0)	3 (33)	0.717
AAGG	2 (40)	0 (0)	1 (20)	2 (40)	0.819
AAGA	2 (20)	4 (40)	1 (10)	3 (30)	0.572
AAAA	0 (%0)	2 (%29)	4 (%57)	1 (%14)	0.368

Genotypes and allotypes of the PVuII (rs2234693) polymorphisms in the ESR1 gene were evaluated in four groups in this study, and the groups were compared. CC genotypes showed no statistically significant difference between groups (p = 0.334). The highest rate of CT genotype was observed in group 1 with 32% as well as in groups 2 and 3 with 20%, but there was no statistically significant difference between the groups (p = 0.651). TT genotypes do not show statistically significant differences between groups (p = 0.363). Genotype and allotype data of ESR1 gene PVuII (rs2234693) polymorphisms are shown in Table 7.

**Table 7:** Genotype and allotype data of ESR1 gene PvuII (Rs2234693) polymorphism Data expressed as mean ± SD. p < 0.05 is considered significant. FSH: Follicle-stimulating hormone; AMH: Anti-mullerian hormone; ESR: Estrogen receptor.

PvuII (Rs2234693)	Group 1: FSH normal, AMH low (n%)	Group 2: FSH high, AMH normal (n%)	Group 3: FSH high, AMH low (n%)	Group 4: FSH normal, AMH normal (n%)	p-value
CC	4 (27)	6 (40)	4 (27)	1 (7)	0.334
CT	14 (32)	9 (20)	9 (20)	12 (27)	0.651
TT	2 (10)	5 (24)	7 (33)	7 (33)	0.363
Allotype					
C	22 (30)	21 (28)	17 (23)	14 (19)	0.529
T	18 (21)	19 (22)	23 (27)	26 (30)	0.592

The GG genotype was observed most frequently with 32% in group 3, 28% in group 4, and 24% in group 2 for the ESR1 XbaI (rs9340799) polymorphism. The lowest rate was observed in group 1 with 16%, but there was no statistically significant difference between the groups (p = 0.706). There is no statistically significant difference in the comparative evaluation of GA genotype between the groups (p = 0.669). The highest rate of AA genotype was observed in group 2 at 42% and in group 3 at 25%, and the lowest values were observed in groups 1 and 4 at 17%, but there was no statistically significant difference between the groups (p = 0.572). Genotype and allotype data of ESR1 gene Xbaı (Rs9340799) polymorphisms are shown in Table 8.

**Table 8:** Genotype and allotype data of ESR1 XbaI (rs9340799) gene polymorphism Data expressed as mean ± SD. p < 0.05 is considered significant. FSH: Follicle-stimulating hormone; AMH: Anti-Mullerian hormone; ESR: Estrogen receptor.

XbaI (rs9340799)	Group 1: FSH normal, AMH low (n%)	Group 2: FSH high, AMH normal (n%)	Group 3: FSH high, AMH low (n%)	Group 4: FSH normal, AMH normal (n%)	p-value
GG	4 (16)	6 (24)	8 (32)	7 (28)	0.706
GA	14 (33)	9 (21)	9 (21)	11 (26)	0.669
AA	2 (17)	5 (42)	3 (25)	2 (17)	0.572
Allotype					
G	22 (24)	21 (23)	25 (27)	25 (27)	0.908
A	18 (27)	19 (28)	15 (22)	15 (22)	0.859

Haplotype analysis of ESR1 gene Rs2234693 and Rs9340799 polymorphisms were analyzed in four groups in this study. No statistically significant difference was observed in the comparative analysis of the groups in the haplotypes analyzed. Haplotype data of ESR1 gene rs2234693 and rs9340799 polymorphisms are given in Table 9.

**Table 9:** Haplotype data of ESR1 gene PvuI (Rs2234693) and XbaI (Rs9340799) polymorphisms Data expressed as mean ± SD. p < 0.05 is considered significant. FSH: Follicle-stimulating hormone; AMH: Anti-Mullerian hormone; ESR: Estrogen receptor.

Haplotype	Group 1: FSH normal, AMH low (n%)	Group 2: FSH high, AMH normal (n%)	Group 3: FSH high, AMH low (n%)	Group 4: FSH normal, AMH normal (n%)	p-value
CCGG	1 (33)	1 (33)	1 (33)	0 (0)	0.999
CCGA	1 (20)	2 (40)	1 (20)	1 (20)	0.896
CCAA	2 (29)	3 (43)	2 (29)	0 (0)	0.867
CTGG	1 (17)	1 (17)	1 (17)	3 (50)	0.572
CTGA	13 (36)	7 (19)	8 (22)	8 (22)	0.485
CTAA	0 (0)	1 (50)	0 (0)	1 (50)	0.999
TTGG	2 (13)	4 (25)	6 (38)	4 (25)	0.572
TTGA	0 (0)	0 (0)	0 (0)	2 (100)	0.999
TTAA	0 (0)	1 (33)	1 (33)	1 (33)	0.999

Genotypes and allotypes of AluI (rs4986938) polymorphisms in the ESR2 gene were evaluated in four groups in this study. For the ESR2 rs4986938 polymorphism, no statistically significant difference was observed in the comparative analysis of the GG genotype in the groups (p = 0.881). GA genotype was highest in group 3 with 31% and lowest in group 2 with 19%. No statistically significant difference was observed between the groups when comparing the GA genotype (p = 0.774). In the comparative analysis of the AA genotype, no statistically significant difference was found between the groups (p = 0.572). In the allotype analysis of rs4986938 polymorphism in the ESR2 gene in four groups, no statistically significant difference was observed in G and A allotypes. Genotype and allotype data of ESR2 gene AluI (Rs4986938) polymorphisms are shown in Table 10.

**Table 10:** Genotype and allotype data of ESR2 gene AluI (Rs4986938) polymorphism Data expressed as mean ± SD. p < 0.05 is considered significant. FSH: Follicle-stimulating hormone; AMH: Anti-Mullerian hormone; ESR: Estrogen receptor.

AluI (rs4986938)	Group 1: FSH normal, AMH low (n%)	Group 2: FSH high, AMH normal (n%)	Group 3: FSH high, AMH low (n%)	Group 4: FSH normal, AMH normal (n%)	p-value
GG	2 (17)	3 (25)	3 (25)	4 (33)	0.881
GA	8 (22)	7 (19)	11 (31)	10 (28)	0.774
AA	10 (31)	10 (31)	6 (19)	6 (19)	0.572
Allotype					
G	12 (20)	13 (22)	17 (28)	18 (30)	0.630
A	28 (28)	27 (27)	23 (23)	22 (22)	0.792

Genotypes and allotypes of ESR2 gene RsaI (Rs1256049) polymorphism in four groups were evaluated in this study. In the genotypic analysis, the GG genotype of this polymorphism was not observed in any group. There was no statistically significant difference in the GA genotype when the groups were compared (p = 0.865). For the AA genotype, no statistically significant difference was observed when comparing the groups (p = 0.982). In the allotype analysis of rs1256049 polymorphism in the ESR2 gene in four groups, no statistically significant difference was observed in G and A allotypes. Genotype and allotype data of ESR2 gene RsaI (Rs1256049) polymorphisms are shown in Table 11.

**Table 11:** Genotype and allotype data of ESR2 gene RsaI (Rs1256049) polymorphism Data expressed as mean ± SD. p < 0.05 is considered significant. FSH: Follicle-stimulating hormone; AMH: Anti-Mullerian hormone; ESR: Estrogen receptor.

RsaI (Rs1256049)	Group 1: FSH normal, AMH low (n%)	Group 2: FSH high, AMH normal (n%)	Group 3: FSH high, AMH low (n%)	Group 4: FSH normal, AMH normal (n%)	p-value
GG	0 (0)	0 (0)	0 (0)	0 (0)	-
GA	5 (33)	3 (20)	4 (27)	3 (20)	0.865
AA	15 (23)	17 (26)	16 (25)	17 (26)	0.982
Allotype					
G	5 (33)	3 (20)	4 (27)	3 (20)	0.865
A	35 (24)	37 (26)	36 (25)	37 (26)	0.990

Haplotype analysis of ESR2 gene rs4986938 and rs1256049 polymorphisms were analyzed in four groups in this study. No statistically significant difference was observed in the comparative analysis of the groups in the haplotypes analyzed. Haplotype data of ESR2 gene rs4986938 and rs1256049 polymorphisms are shown in Table 12.

**Table 12:** Haplotype data of ESR2 gene AluI (Rs4986938) and RsaI (Rs1256049) polymorphisms Data expressed as mean ± SD. p < 0.05 is considered significant. FSH: Follicle-stimulating hormone; AMH: Anti-Mullerian hormone; ESR: Estrogen receptor.

Haplotype	Group 1: FSH normal, AMH low (n%)	Group 2: FSH high, AMH normal (n%)	Group 3: FSH high, AMH low (n%)	Group 4: FSH normal, AMH normal (n%)	p-value
GGGG	0 (0)	0 (0)	0 (0)	0 (0)	-
GGGA	0 (0)	0 (0)	0 (0)	1 (100)	-
GGAA	2 (18)	3 (27)	3 (27)	3 (27)	0.965
GAGG	0 (0)	0 (0)	0 (0)	0 (0)	-
GAGA	0 (0)	1 (33)	1 (33)	1 (33)	0.999
GAAA	8 (24)	6 (18)	10 (30)	9 (27)	0.787
AAGG	0 (0)	0 (0)	0 (0)	0 (0)	-
AAGA	5 (45)	2 (18)	3 (27)	1 (9)	0.364
AAAA	5 (24)	8 (38)	3 (14)	5 (24)	0.488

## Discussion

Nowadays, there is an increasing number of babies born as a result of assisted reproduction technology (ART). Various markers have been used to predict ovarian response when planning the treatment protocol, with FSH and AMH being the most popular biochemical tests. Accurate individual interpretation of FSH and AMH is important in predicting in vitro fertilization (IVF) success. Currently, research is ongoing to investigate whether genetic factors may affect this individual assessment.

Studies in different populations have shown that FSHR and ER polymorphisms are associated with ovarian response [18-20]. Boudjenah et al. found no association of Asn680Ser, Ala189Val, Thr449Ile, and Ile160Thr polymorphisms on the FSHR gene with poor ovarian response to exogenous gonadotropins [20]. Alviggi et al. showed that patient groups with the Ser/Ser genotype were less sensitive to exogenous gonadotropins [13]. König et al. compared the ovarian response to exogenous gonadotropins in three groups with Ser/Ser, Asn/Ser, and Asn/Asn genotypes and found that Ser/Ser genotype was significantly associated with poor response to treatment [14]. In our study, genotypes, allotypes, and haplotypes of The307Ala (rs6165) and Asn680Ser (rs6166) polymorphisms of FHSR gene polymorphisms were analyzed. As a result of our research, no statistically significant difference was found in terms of these polymorphisms in the comparative analysis of the groups.

The ESR1 gene is responsible for encoding the ESR protein, which plays an important role in the proliferation of granulosa cells and the regulation of folliculogenesis. Although SNPs on the ESR1 gene have been shown to cause poor ovarian response, current data are still conflicting [17,21]. De Mattos et al. showed a direct association between SNPs on the ESR1 gene and poor ovarian response [21]. There was no significant association between polymorphisms in the ESR1 gene and poor ovarian response in the study by Li et al. [17]. In our study, genotypes and allotypes of ESR1 gene PVuII (rs2234693) polymorphism, XbaI (rs9340799) polymorphism, and haplotypes of these two polymorphisms were analyzed in 80 patients grouped according to FSH and AMH values. In addition, genotypes and allotypes of ESR2 gene AluI (rs4986938) polymorphism and genotypes, allotypes, and haplotypes of RsaI (rs1256049) polymorphism were investigated. As a result of our research, no statistically significant difference was found in terms of these polymorphisms in the comparative analysis of the groups.

The effect of obesity on the testing of ovarian reserve is not clear [22]. However, obesity may increase adipokines or other inflammatory markers in the ovaries, leading to a decrease in the follicular pool [23,24]. In a study by Marsh et al. investigating the relationship between BMI and AMH, it was found that AMH decreased as BMI increased [24]. A meta-analysis conducted in 2018 showed that AMH concentrations in obese women were significantly lower than those in non-obese women [25]. In our study, group 1 had the highest BMI (27.95 ± 3.137), and group 2 had the lowest BMI (25.05 ± 3.41). In the comparative analysis of BMI between groups, a statistically significant difference was observed between groups 1 and 2. A statistically significant difference was also observed in the comparative analysis of BMI values between groups 2 and 3. This significant difference draws attention to the association between obesity and low AMH levels.

AMH and FSH levels reflect the ovarian reserve at different stages of the follicular process; the antral and post-antral follicular developments are reflected by FSH, whereas post-primordial pre-antral and early antral follicular developments are reflected by AMH. In recent years, AMH has been preferred to baseline FSH because it can be measured at any time during the menstrual cycle and has a higher prognostic value both as a marker of ovarian reserve and in predicting pregnancy rate, according to studies in patients undergoing IVF treatment [26]. Salama et al. showed that in women under 35 years of age, basal FSH levels were more strongly associated with follicle number and number of oocytes retrieved, whereas in women over 35 years of age, AMH rather than FSH was more strongly associated with the same outcomes [27].

In a study of 361 women with AMH levels < 0.5 ng/ml, Revelli et al. found that young patients with very low AMH levels still had a reasonable chance of successful pregnancy with IVF [28]. Ligon et al. evaluated discordant and concordant values of AMH and FSH on live birth rates and IVF cycle cancellation rates. The live birth rate in patients with normal AMH and elevated FSH was higher than that in patients with low AMH and normal FSH (39 vs. 26%). The live birth rate in patients with normal AMH and normal FSH (concordant) was higher than that in any other group (44%). In addition, the IVF cycle cancellation rate in patients with normal AMH and FSH was lower than that in other groups (4%), and this rate was higher in patients with elevated FSH and low AMH compared to other groups (30%) [29]. This difference might be caused by several factors including different study populations with different genetic and environmental backgrounds, which could lead to a different ovarian biological age compared to chronological age. Multiple genetic factors have been investigated to explain this genetic basis. The association between single SNPs and diseases has long been known, and it has been shown that some SNPs have correlated with an increased risk of endometriosis in populations with different ethnicities [2-4]. Pharmacogenetic analysis is very important in increasing the specificity and sensitivity of a biomarker in ovarian reserve assessment.

This study has several limitations. First, it was conducted in a single center with a limited number of patients. Second, the analysis of a single ethnic group may have been inadequate in terms of study design. Finally, the wide age range may have affected our results.

## Conclusions

The results of this study showed that there was no significant difference in FSHR, ESR1, and ESR2 SNPs between FSH-AMH discordant women with low ovarian reserve and women with normal ovarian reserve. In groups with low AMH levels, an increase in BMI was observed. Further studies are needed to determine the genetic basis of FSH-AMH discordance. This may have implications for personalized infertility treatment.
